# Association between fatty liver index and blood coagulation markers: a population-based study

**DOI:** 10.1186/s12944-023-01854-8

**Published:** 2023-06-29

**Authors:** Maximilian Iglesias Morcillo, Dennis Freuer, Anette Peters, Margit Heier, Daniel Teupser, Christine Meisinger, Jakob Linseisen

**Affiliations:** 1 Epidemiology, University of Augsburg, University Hospital Augsburg, Stenglinstr. 2, Augsburg, 86156 Germany; 2 Institute of Epidemiology, Helmholtz Zentrum München – German Research Center for Environmental Health (GmbH), Neuherberg, 85764 Germany; 3 Chair of Epidemiology, Institute for Medical Information Processing, Biometry and Epidemiology, Medical Faculty, Ludwig-Maximilians-Universität München, Munich, 81377 Germany; 4 German Center for Diabetes Research (DZD), Munich-Neuherberg, 85764 Germany; 5 KORA Study Centre, University Hospital Augsburg, Augsburg, 86156 Germany; 6 Institute of Laboratory Medicine, Medical Faculty, Ludwig-Maximilians-Universität München, Munich, 81377 Germany; 7 Institute for Medical Information Processing, Biometry and Epidemiology, Medical Faculty, Ludwig-Maximilians-Universität München, Munich, 81377 Germany

**Keywords:** Fatty liver index, Coagulation, Hemostatic factors, Hepatic steatosis, KORA

## Abstract

**Background:**

Population-based studies investigating the association between blood coagulation markers and non-alcoholic fatty liver disease (NAFLD) are rare. Thus, we aimed to investigate the relationship between the Fatty Liver Index (FLI) as a measure of hepatic steatosis and plasma concentrations of antithrombin III, D-dimer, fibrinogen D, protein C, protein S, factor VIII, activated partial thromboplastin time (aPTT), quick value and international thromboplastin time (INR) in the general population.

**Methods:**

After the exclusion of participants with anticoagulative treatment, 776 participants (420 women and 356 men, aged 54–74 years) of the population-based KORA Fit study with analytic data on hemostatic factors were included in the present analysis. Linear regression models were used to explore the associations between FLI and hemostatic markers, adjusted for sex, age, alcohol consumption, education, smoking status, and physical activity. In a second model, additional adjustments were made for the history of stroke, hypertension, myocardial infarction, serum non-HDL cholesterol levels, and diabetes status. In addition, analyses were stratified by diabetes status.

**Results:**

In the multivariable models (with or without health conditions), significantly positive associations with FLI were obtained for plasma concentrations of D-dimers, factor VIII, fibrinogen D, protein C, protein S, and quick value, while INR and antithrombin III were inversely associated. These associations were weaker in pre-diabetic subjects and largely disappeared in diabetic patients.

**Conclusion:**

In this population-based study, an increased FLI is clearly related to changes in the blood coagulation system, possibly increasing the risk of thrombotic events. Due to a generally more pro-coagulative profile of hemostatic factors, such an association is not visible in diabetic subjects.

## Background

Non-alcoholic fatty liver disease (NAFLD) is highly prevalent and includes a spectrum of abnormalities ranging from simple steatosis to non-alcoholic steatohepatitis (NASH) to cirrhosis. With a prevalence of around 25% worldwide, it has become the most frequent chronic liver disease in recent decades [1]. Fatty liver, also called steatotic hepatitis, is a common disease in industrialized nations [1, 2]. NAFLD often remains undiagnosed for a long time, and there is still no specific medication available [2]It is closely related to type 2 diabetes mellitus (T2DM) or impaired fasting glucose, dyslipidemia, obesity, and hypertension. Excessive dietary energy intake and progressive obesity lead to the accumulation of body fat stores, including lipid deposits in the liver, resulting in alterations of glucose and lipid metabolism, and inflammation. Thus, environmental factors, including an energy-dense diet and low physical activity, in combination with genetic factors, promote the development of metabolic derangements and eventually NAFLD. Without medical intervention and a comprehensive change in lifestyle and body fat mass, fatty liver disease may develop into fatty liver inflammation (steatohepatitis), liver fibrosis, cirrhosis, and eventually liver cancer [2–5]. However, this is not a strict sequence as, e.g., fibrosis may already occur at the early stages of NAFLD [6]In addition, in subjects with NAFLD the risk of cardiovascular diseases, including heart attack and type 2 diabetes, considerably increases [7].

Bedogni and coworkers were the first to describe a simple formula for calculating the so-called Fatty Liver Index (FLI). By including data on waist circumference, body mass index, serum triglycerides and the liver enzyme gamma-glutamyl transferase, they were able to develop and validate a score [8]. This simple tool can be used to determine the likelihood of fatty liver disease quickly and easily. However, it lacks the precision of histologic analysis of liver biopsies. Liver biopsy analysis remains the diagnostic gold standard, but its use is limited by high costs and procedural risk [9]. The high incidence of NAFLD makes routine biopsies both impractical and impossible in all patients [10] Imaging techniques, including ultrasonography of the liver, are also used to analyze fat accumulation in the liver, and the FLI was validated against the results of liver ultrasonography. Also, the application of imaging techniques requires specific devices and trained personnel [10] Among many other functions of the liver, this organ also produces most of the coagulation markers and thus plays a key role in blood coagulation [11]; abnormalities of the liver, including a high liver cell fat content, may result in modifications of liver-derived hemostatic factors and impact the blood coagulation system [12]. Blood clotting markers that initiate or drive blood clotting or lead to the formation of a clot, such as fibrinogen D or factor VIII, are mainly formed in the liver. In addition, proteins and enzymes such as antithrombin III, protein C and protein S are also produced in the liver and play a major role in inhibiting blood clotting, thus acting as natural anticoagulants [11]. Simplified speaking, blood clotting is triggered by the ladder-like activation of individual enzymes, which, through their activation, trigger a cascade that results in the formation of a thrombus [13].

The prevalence of insulin resistance is high in NAFLD and even higher in patients with NASH [14]. As there is an indication that treatment of prediabetic patients also leads to the normalization of coagulation markers [15], the relationship between glucose tolerance status and FLI needs exploration. Thus, the purpose of this study was to investigate the relationship between FLI and a large set of clinically relevant blood coagulation markers in the general population as FLI could be used as a screening tool to identify NAFLD (followed by clinical characterization, and eventually intervention). Additionally, the situation in diabetic and prediabetic subjects will be explored. If an influence of the FLI on hemostatic factors - and thus the coagulation cascade - can be confirmed at the population level, this would provide further support for preventive measures to decrease the liver fat content and thus avoid the development of liver fibrosis and metabolic (and cardio-vascular) complications.

## Materials and methods

### Study sample

The KORA (Cooperative Health Research in the region of Augsburg) project continued the MONICA (Monitoring trends and determinants in cardiovascular disease) project in 1996. Together, they had the objective of investigating the health status of the adult inhabitants of the Augsburg region, with a special focus on cardiovascular diseases, lung diseases, diabetes, and environmental exposure factors [16]. Four cross-sectional surveys, KORA survey 1 (S1) to KORA survey 4 (S4), were carried out in a period from 1984 to 2001, and 18,000 participants aged 25–74 years were recruited and their health status was assessed; several follow-up studies were conducted [17]. In the 2018–2019 KORA Fit Follow-up Study, all living participants of the KORA cohort born between 1945 and 1964 were enrolled to get information on the health status of all middle-agers in the full KORA cohort. All living KORA S4 participants who had participated in KORA Fit and for whom citrated plasma samples were available were eligible for the present analyses. A total of 776 participants, 420 women and 356 men, were considered after the exclusion of participants undergoing anticoagulative treatment (n = 29). All study participants gave written informed consent. The study protocol was approved by the Ethics Committee of the Bavarian Chamber of Physicians (KORA Fit EC No. 17040) and conducted according to the data protection requirements. The investigations were carried out in accordance with the Declaration of Helsinki.

### Data collection

Participants of the KORA Fit study were interviewed by certified and specially trained personnel in a standardized interview about lifestyle factors, such as smoking behavior, physical activity, alcohol consumption, preexisting diseases, medication intake and socio-demographic variables. Alcohol consumption was calculated in grams per day assessed for the previous workday and the previous weekend. Education was recorded in completed school years (≤ 12 years and > 12 years). Physical activity was estimated using two separate four-item interview questions asking about the time per week spent on leisure-time sports (including cycling) in summer and winter. The winter and summer responses were combined to create one variable of leisure-time physical activity. First, participants were classified into one of four categories for frequency of leisure time activity: ‘No activity’ was defined as no sport in either summer or winter; ‘Low activity’ was irregular participation in sport for approximately 1 hour per week in at least one season; ‘Moderate activity’ was regular participation in sport for approximately 1 hour per week in at least one season; ‘High activity’ was regular participation in sport in both summer and winter for more than 2 h per week in both seasons. The previous existence of myocardial infarction and stroke (yes/no) was recorded but not verified against medical records. Smoking behavior was recorded as current, former and never smoker. Persons aware of having hypertension, taking antihypertensive medication, and/or having blood pressure values of 140/90 mmHg or higher at baseline were defined as actual hypertensives (yes/no). Diabetes status was assessed by HbA1c (%) and serum fasting glucose (mg/dl) and categorized into five subgroups: normal status, prediabetes (fasting plasma glucose levels: ≥100- <126 and/or HbA1c: ≥5.7- <6.5), newly diagnosed diabetes (fasting plasma glucose levels: ≥126 or HbA1c: ≥6.5), known diagnosis of diabetes 2, and other type or vague status. Body mass index was calculated as weight in kilograms divided by height in square meters [18]. Waist circumference was measured to the nearest 0.1 cm at the midpoint between the lower margin of the least palpable rib and the top of the iliac crest using stretch-resistant tape [19]. The interview process and all measurements were done according to the standard operating procedures; the devices used were regularly calibrated according to the manufacturer’s advice. Study nurses were trained according to SOPs, certified and regularly re-certified for each examination module. Quality assurance measures were applied throughout the field phase, and for the final data control, all laid down in the quality assurance report. Detailed information on the data collection, examination procedures and variable definitions in the KORA studies is described in detail elsewhere [20].

### Laboratory measurements

All hemostatic measurements were performed in citrated plasma collected after an overnight fasting state. Samples were processed immediately (centrifugation 10 min at 15 °C), aliquoted and stored at -80 °C.

Antithrombin III activity (reference value: 83–118%) was measured by chromogenic activity assay (Innovance Antithrombin, SCS cleaner, Siemens Eschborn). D-Dimers were determined by means of a particle-enhanced immunoturbidimetric assay (ref. value: <500 μg/dL; Innovance D-Dimer Kit, Siemens Eschborn). Faktor VIII activity (ref. value: 70–150%) was analyzed using photometry (coagulation factor VIII deficient plasma, Pathromtin SL, CaCl2, Siemens Eschborn). Fibrinogen (ref. value: 210–400 mg/dL) was quantified photometrically and turbidimetrically (Multifibern U, Siemens Eschborn). The parameters of aPTT (ref. value: 26–36 s; Pathromtin SL, CaCl Lösung, Actin FS, Siemens Eschborn), protein C and S activity (ref. value for protein C: 70–140%; ref. value for protein S: 73–130% for men, 52–126% for women; Berichrom Protein C, Siemens Healthcare; Hemoclot Protein S, OVB-Puffer, CaCl2, SCS-Cleaner) and quick value (ref. value: 82–125%; Thromborel S Siemens Eschborn) were determined photometrically. INR (ref. value: 0.9–1.15) was calculated from the prothrombin ratio (Thromborel S, Siemens Eschborn) by dividing the thromboplastin time of the subject by the thromboplastin time of normal plasma squared with the International Sensitivity Index (ISI) according to the WHO [21]. Serum triglycerides (ref. value: 0-200 mg/dl) were determined by an enzymatic color test (Hoffman-La Roche AG Basel/Switzerland) on a Cobas 8000 c702 (Hoffman-La Roche AG Basel/Switzerland). Serum total cholesterol levels (ref. value: 0-200 mg/dL) and HDL cholesterol (ref. value: >45 mg/dL) were analyzed enzymatically (Cobas 8000 c702 Roche chemistry analyzer; Hoffman-La Roche AG, Basel/Switzerland). Non-HDL cholesterol was built by subtracting HDL cholesterol from total cholesterol. Gamma-glutamyl transferase (GGT) was analyzed according to the IFCC method (Hoffmann-La Roche AG Basel/Switzerland), and serum glucose was quantified using the hexokinase method (Hoffmann-La Roche AG Basel/Switzerland). For the determination of glycosylated haemoglobin (HbA1c), an HPLC method with UV detection (Variant II Turbo Hemoglobin Test System; Biorad Hercules, California/USA) was used.

Reference values of all blood coagulation markers were taken from Hospital Augsburg, and all other parameters were measured at the clinical laboratory of the University Hospital of the Ludwig-Maximilians University in Munich (Klinikum Großhadern). All measurement procedures were performed and controlled according to standardized protocols by trained laboratory personnel.

The Fatty Liver Index was calculated according to the formula of Bedogni et al. [8]:

$$\begin{array}{l}FLI = \left( {{e^{\scriptstyle0.953 \times {\rm{log}}\left( {triglycerides} \right) + 0.139 \times BMI + 0.718\hfill\atop\scriptstyle \times {\rm{log}}\left( {GGT} \right) + 0.053 \times waist\,\,circumference - 15.745\hfill}}} \right)\\\div \left( {1 + {e^{\scriptstyle0.953 \times {\rm{log}}\left( {triglycerides} \right) + 0.139 \times BMI + 0.718\hfill\atop\scriptstyle \times {\rm{log}}\left( {GGT} \right) + 0.053 \times waist\,\,circumference - 15.745\hfill}}} \right)\\\times 100\end{array}$$

FLI was categorized into three groups. Values between 0 and 29 rule out fatty liver disease and thus hepatic steatosis, values between 30 and 59 are defined as unclear, and a score of 60 and higher indicates the presence of fatty liver disease (hepatic steatosis).

### Statistical analyses

Continuous variables were presented by means and standard deviation, and nonnormally distributed variables were described by median and interquartile range. The Shapiro‒Wilk test was applied to test for normal distribution. The Kruskal‒Wallis test was used to test for differences between the medians of three or more independent groups.

Linear regression models were applied to investigate associations between FLI (continuous) as an exposure variable and blood coagulation markers as dependent variables. The models were adjusted for age (continuous), alcohol consumption (continuous), sex (male; female), education (< 12 years; ≥12 years), smoking status (never; former; current) and physical activity (≥ 2 h/week; 1 h/week; <1 h/week; (almost) no activity). In a second model, additional adjustments were made for stroke (yes/no), hypertension (yes/no), myocardial infarction (yes/no), serum non-HDL cholesterol (continuous) and diabetes status (normal; prediabetes; newly diagnosed; known type of diabetes; other type or vague). We tested for interaction effects with diabetes status by including a multiplicative term in the fully adjusted models. Stratified analysis by diabetes status (normal; prediabetes; newly diagnosed and known diabetes) was conducted to describe differential associations as indicated by significant interaction results. All model assumptions were ensured, including homoscedasticity. Multicollinearity and autocorrelation were assessed using the variance inflation factor (VIF) and the Durbin-Watson statistic, respectively. A value of *P* < 0.05 was considered statistically significant. All analyses were performed with IBM SPSS 29 (IBM Inc., Armonk, New York; USA).

## Results

Table 1 shows the baseline characteristics of the 776 participants included in the analyses, stratified by sex. The mean age was the same for both males and females (mean total: 62.6; SD: 5.6). Men (mean: 60.0; SD: 27.6) showed a higher average FLI than women (mean: 39.1; SD: 30.1). Thus, 201 (56.5%) men were regarded as having fatty liver disease. Among women, only 125 (29.7%) were classified as subjects with fatty liver disease. The proportion of females with normal glucose tolerance (233; 55.5%) was higher than that of males (144; 40.5%). Accordingly, the prevalence of prediabetes or diabetes was higher in men than in women.

**Table 1:** Characteristics of the study participants^a^

		Total (n = 776)		Males (n = 356)		Females (n = 420)	
				Mean	SD		
Age [years]		62.6	5.6	62.6	5.8	62.6	5.5
FLI		48.7	30.8	60.0	27.6	39.1	30.1
BMI [kg/m²]		27.9	4.8	28.4	4.2	27.4	5.2
Waist circumference [cm]		94.0	13.8	100.5	12.2	88.4	12.6
Gamma glutamyl transferase [U/l]		35.5	40.2	42.7	34.8	29.4	43.4
Glutamate oxalate transaminase [U/l]		26.1	12.9	27.6	10.2	24.9	14.6
Glutamate pyruvate transaminase [U/l]		28.2	16.0	32.3	14.7	24.7	16.4
Total cholesterol [mg/dL]		212.1	40.2	201.4	39.8	221.1	38.3
HDL cholesterol [mg/dL]		64.3	18.9	55.3	15.2	71.9	18.4
Non-HDL cholesterol [mg/dL]		147.8	39.1	146.1	39.6	149.3	38.6
		Median (25th – 75th percentile)					
Alcohol consumption^b^ [g/d]		5.7 (0.0;22.9)		5.7(0.0;21.5)		6.7 (0.0;22.9)	
Triglycerides [mg/dL]		106.0 (77.0;148.0)		114.0 (80.5;164.0)		100.0 (72.8;136.0)	
				N	%		
FLI	<30, no hepatic steatosis	287	37.0%	73	20.5%	214	51.0%
	30–59, intermediate status	163	21.0%	82	23.0%	81	19.3%
	≥ 60, hepatic steatosis	326	42.0%	201	56.5%	125	29.7%
Education [years]	≤ 12 years	471	61.0%	198	56.0%	273	63.0%
	> 12 years	305	39.0%	158	44.0%	147	37.0%
Physical activity	high activity (≥ 2 h/week)	284	37.0%	133	37.5%	151	36.0%
	moderate activity (1 h/week)	264	34.0%	115	32.5%	149	35.5%
	low activity (< 1 h/week)	94	12.0%	50	14.0%	44	10.5%
	(almost) no activity	134	17.0%	58	16.0%	76	18.0%
Smoking	current smoker	106	14.0%	53	15.0%	53	13.0%
	former smoker	335	43.0%	176	49.5%	159	38.0%
	never smoker	335	43.0%	127	35.5%	208	49.0%
Hypertension^b^	yes	361	46.5%	196	55.0%	165	39.0%
	no	414	53.5%	159	45.0%	255	61.0%
Myocardial infarction	yes	22	2.8%	18	5.0%	4	1.0%
	no	754	97.2%	338	95.0%	416	99.0%
Stroke	yes	18	2.3%	12	3.4%	6	1.5%
	no	758	97.7%	344	96.6%	414	98.5%
Glucose tolerance status	normal	377	48.6%	144	40.5	233	55.5%
	prediabetes	303	39.0%	159	44.7%	144	34.3%
	newly diagnosed diabetes	16	2.0%	12	3.4%	4	0.9%
	known type 2 diabetes	68	8.8%	37	10.4%	31	7.4%
	other type of diabetes or vague	12	1.6%	4	1.0%	8	1.9%

^a^ After exclusion of individuals with anticoagulation therapy (n = 29); ^b^ n = 775, 1 = missing

Median plasma concentrations of coagulation markers are described in Table 2, stratified by FLI categories and glucose tolerance status. Overall, the mean concentrations of blood coagulation markers were in a normal range, except for protein S. Protein S concentrations were elevated for the total sample in the intermediate FLI category (median: 131.3; IQR: 107.8;150.3) and in subjects with hepatic steatosis (median: 132.1; IQR: 112.9; 153.0). When the models were stratified according to glucose tolerance status, the same results were found in all categories, namely, an increased protein S level in the intermediate status and in hepatic steatosis. In the case of newly diagnosed and known diabetes, an increased level of D-dimer was also found in the intermediate status group of FLI (median: 511.5; IQR: 429.0;874.0).

**Table 2:** Blood coagulation markers (median; IQR) according to Fatty Liver Index (FLI) categories in all participants^a^ and stratified by glucose tolerance status

Total sample (n = 776)	FLI^b^			
	no hepatic steatosis [FLI < 30]	intermediate status [FLI 30–59]	hepatic steatosis [FLI ≥ 60]	*P* value^e^
	n = 287	n = 163	n = 326	
Antithrombin III [mg/dL]	105.5 (99.0;112.5)	102.1 (96.0;107.9)	98.9 (92.5;106.0)	< 0.001
D-dimers[μg[l/	367.0 (276.0;506.0)	408.0 (330.0;557.0)	438.5 (334.0;624.0)	< 0.001
Faktor VIII [%]	116.8 (93.8;139.3)	117.3 (94.1;137.9)	125.8 (103.4;151.4)	< 0.001
Fibrinogen D [mg/dL]	280.6 (249.1;316.0)	298.4 (265.4;336.1)	307.4 (268.5;345.4)	< 0.001
aPTT [s]^c^	30.7 (28.6;33.1)	30.8 (29.1;32.8)	30.6 (28.6;32.8)	0.671
Protein C [%]	122.5 (111.7;136.8)	124.9 (112.0;140.9)	124.2 (111.8;140.3)	0.207
Protein S [%]	115.9 (98.8;132.9)	131.3 (107.8;150.3)	132.1 (112.9;153.0)	< 0.001
Quick value [%]	107.3 (100.2;112.6)	109.2 (103.1;115.5)	109.4 (103.7;115.7)	0.001
INR^d^	1.0 (0.9;1.0)	1.0 (0.9;1.0)	1.0 (0.9;1.0)	0.001
**Normal glucose tolerance (n = 377)**	n = 194	n = 78	n = 105	
Antithrombin III [mg/dL]	105.5 (99.1;112.5)	102.4 (96.2;108.7)	100.7 (95.2;107.5)	< 0.001
D-dimers[μg[l/	348.0 (270.0;470.0)	379.0 (296.0;607.0)	429.0 (332.0;602.0)	< 0.001
Faktor VIII [%]	116.1 (91.7;139.0)	113.0 (95.6;134.8)	123.5 (100.3;143.5)	0.134
Fibrinogen D [mg/dL]	277.3 (248.8;305.6)	297.5 (260.2;334.6)	302.3 (262.3;342.8)	< 0.001
aPTT [s]^c^	30.7 (28.7;33.1)	30.08 (29.1;33.3)	31.3 (29.0;33.3)	0.644
Protein C [%]	122.4 (109.6;136.5)	127.8 (114.8;140.9)	127.5 (113.7;139.2)	0.055
Protein S [%]	111.5 (96.7;129.6)	132.2 (106.6;152.5)	132.1 (110.1;162.3)	< 0.001
Quick value [%]	106.4 (99.7;111.6)	109.5 (103.2;115.8)	108.9 (103.0;117.7)	0.006
INR^d^	1.0 (0.9;1.0)	1.0 (0.9;1.0)	1.0 (0.9;1.0)	0.005
**Prediabetes (n = 303)**	n = 84	n = 72	n = 147	
Antithrombin III [mg/dL]	105.6 (97.0;110.4)	100.9 (95.4;106.2)	98.5 (91.2;105.3)	< 0.001
D-dimers[μg[l/	414.5 (333.0;562.0)	415.5 (349.0;516.0)	430.0 (326.0;606.0)	0.871
Faktor VIII [%]	116.8 (95.9;139.4)	119.0 (93.4;142.1)	125.5 (101.7;150.9)	0.056
Fibrinogen D [mg/dL]	296.3 (250.3;347.5)	296.8 (269.7;340.0)	310.3 (271.0;341.6)	0.468
aPTT [s]^c^	31.1 (28.6;33.6)	30.8 (28.8;32.8)	30.1 (28.2;32.6)	0.201
Protein C [%]	123.5 (112.4;137.5)	122.3 (111.4;133.4)	123.5 (111.6;140.1)	0.959
Protein S [%]	123.8 (105.1;140.7)	130.2 (109.5;148.9)	134.2 (113.9;150.2)	0.050
Quick value [%]	108.8 (102.8;115.0)	108.8 (101.9;114.8)	110.5 (104.6;115.5)	0.381
INR^d^	1.0 (0.9;1.0)	1.0 (0.9;1.0)	0.9 (0.9;1.0)	0.396
**Diabetes newly diagnosed and known (n = 84)**	n = 6	n = 10	n = 68	
Antithrombin III [mg/dL]	109.8 (98.3;121.0)	104.4 (97.8;109.7)	96.3 (91.4;104.0)	0.038
D-dimers[μg[l/	419.0 (289.0;552.0)	511.5 (429.0;874.0)	499.5 (374.5;716.0)	0.486
Faktor VIII [%]	137.1 (97.4;177.7)	122.3 (115.8;139.8)	136.6 (114.1;162.5)	0.355
Fibrinogen D [mg/dL]	299.9 (274.8;356.5)	304.0 (284.0;318.3)	313.2 (277.1;349.9)	0.786
aPTT [s]^c^	28.4 (25.5;30.4)	31.1 (29.6;32.9)	30.6 (28.8;32.7)	0.108
Protein C [%]	124.3 (119.7;133.1)	124.6 (114.3;141.1)	120.9 (110.6;142.0)	0.919
Protein S [%]	104.9 (94.4;137.3)	134.5 (122.3;156.7)	130.9 (112.8;146.4)	0.242
Quick value [%]	113.4 (108.4;121.0)	111.1 (103.6;116.1)	108.7 (101.6;115.7)	0.297
INR^d^	0.9 (0.9;1.0)	0.9 (0.9;1.0)	1.0 (0.9;1.0)	0.286

^a^ After exclusion of individuals with anticoagulation therapy (n = 29); ^b^ Fatty Liver Index; ^c^ activated partial thromboplastin time ^d^ international normalized ratio ^e^ Kruskal‒Wallis test

Table 3 shows the results of the linear regression models exploring the association between FLI and blood coagulation markers in the total sample. In the models adjusted for age, alcohol consumption, sex, education, smoking status, and physical activity, significant positive associations were detected for D-dimers, factor VIII, fibrinogen D, protein C, protein S and quick value; inverse associations were obtained for antithrombin III and INR, and aPTT was not significantly associated with FLI (model 1). Similar results were obtained with the second adjustment model, with all hemostatic factors showing significant relationships, but aPTT was also significantly inversely associated with FLI (model 2).

**Table 3:** Association between the Fatty Liver Index (FLI) and blood coagulation parameters in all study participants^a^

	Model 1^b^			Model 2^c^		
	𝛃 estimate	95% confidence interval	*P* value	𝛃 estimate	95% confidence interval	*P* value
Antithrombin III [mg/dl]	-0.069	-0.095; -0.042	< 0.001	-0.080	-0.109; -0.051	< 0.001
Ln D-dimers	0.003	0.002; 0.004	< 0.001	0.002	0.001; 0.004	0.002
Faktor VIII [%]	0.232	0.144; 0.321	< 0.001	0.215	0.115; 0.314	< 0.001
Fibrinogen D [mg/dL]	0.418	0.260; 0.576	< 0.001	0.373	0.196; 0.551	< 0.001
aPTT [s]^d^	-0.008	-0.017; 0.000	0.055	-0.010	-0.020; -0.001	0.032
Protein C [%]	0.108	0.064;0.153	< 0.001	0.083	0.035; 0.132	< 0.001
Protein S [%]	0.240	0.155; 0.325	< 0.001	0.184	0.089; 0.279	< 0.001
Quick value [%]	0.054	0.030; 0.078	< 0.001	0.048	0.021; 0.074	< 0.001
INR^e^	-0.032	-0.048; − 0.017	< 0.001	-0.031	-0.048; -0.014	< 0.001

^a^ After exclusion of individuals with anticoagulation therapy (n = 29); ^b^ dependent variables: blood coagulation markers, exposure variable: Fatty Liver Index, adjusted for age, alcohol consumption, sex, education, smoking and physical activity; ^c^ dependent variables: blood coagulation markers, exposure variable: Fatty Liver Index, additionally adjusted for stroke, hypertension, myocardial infarction, non HDL cholesterol, diabetes; ^d^ activated partial thromboplastin time; ^e^ international normalized ratio, INR times 100

Testing for interaction between FLI and diabetes status revealed several significant results. When stratifying the analysis by diabetes status, the significant associations between FLI and coagulation parameters observed in non-diabetic subjects were not observed in diabetic subjects (except for antithrombin III in model 1). Subjects with normal glucose tolerance (Table 4) showed almost the same results as reported for the whole sample (Table 3). In prediabetic subjects, significant positive associations were obtained for factor VIII, fibrinogen D, protein C and protein S. Antithrombin III and aPTT were significantly inversely associated (model 1). In the second adjusted model (model 2), antithrombin III, factor VIII and aPTT were the only significant coagulation markers, with aPTT being inversely associated.

**Table 4:** Association between the Fatty Liver Index (FLI) and blood coagulation parameters in study participants^a^ stratified by glucose tolerance status

	Model 1^b^					Model 2^c^		
	𝛃 estimate	95% confidence interval			*P* value	𝛃 estimate	95% confidence interval	*P* value
**Normal glucose tolerance**								
Antithrombin III [mg/dL]	-0.042	-0.080; -0.004			0.032	-0.061	-0.103; -0.019	0.004
Ln D-dimers	0.003	0.001; 0.005			0.003	0.004	0.002; 0.006	< 0.001
Faktor VIII [%]	0.206	0.068; 0.343			0.003	0.239	0.087; 0.392	0.002
Fibrinogen D [mg/dL]	0.544	0.303; 0.785			< 0.001	0.502	0.235; 0.769	< 0.001
aPTT [s]^d^	0.001	-0.013; 0.012			0.940	-0.009	-0.023; 0.005	0.203
Protein C [%]	0.163	0.098; 0.229			< 0.001	0.116	0.045; 0.187	0.002
Protein S [%]	0.305	0.177; 0.433			< 0.001	0.215	0.074; 0.355	0.003
Quick value [%]	0.082	0.047; 0.117			< 0.001	0.076	0.037; 0.115	< 0.001
INR^e^	-0.049	-0.070; − 0.028			< 0.001	-0.045	-0.069; -0.022	< 0.001
**Prediabetes**								
Antithrombin III [mg/dL]	-0.072	-0.113; -0.031			< 0.001	-0.097	-0.139; -0.054	< 0.001
Ln D-dimers	0.001	-0.001; 0.003			0.251	0.001	-0.001; 0.003	0.528
Faktor VIII [%]	0.279	0.132; 0.427			< 0.001	0.251	0.097; 0.405	0.002
Fibrinogen D [mg/dL]	0.210	-0.055; 0.474			< 0.001	0.169	-0.112; 0.450	0.237
aPTT [s]^d^	-0.017	-0.031; -0.003			0.021	-0.018	-0.033; -0.002	0.023
Protein C [%]	0.078	0.007; 0.149			0.032	0.055	-0.018; 0.129	0.141
Protein S [%]	0.149	0.011; 0.287			0.035	0.127	-0.017; 0.272	0.083
Quick value [%]	0.030	-0.005; 0.065			0.096	0.032	-0.005; 0.070	0.088
INR^e^	-0.018	-0.039; 0.003			0.089	-0.020	-0.042; 0.002	0.077
**Diabetes, newly diagnosed and known**								
Antithrombin III [mg/dL]	-0.175		-0.318; -0.032	0.017		-0.120	-0.275; 0.034	0.125
Ln D-dimers	0.003		-0.003; 0.008	0.306		0.003	-0.003; 0.009	0.319
Faktor VIII [%]	0.152		-0.179; 0.483	0.364		0.114	-0.250; 0.479	0.533
Fibrinogen D [mg/dL]	0.160		-0.437; 0.758	0.594		0.320	-0.330; 0.971	0.329
aPTT [s]^d^	0.002		-0.035; 0.040	0.903		-0,002	-0.043; -0.039	0.919
Protein C [%]	-0.019		-0.241;0.203	0.865		0.007	-0.228; 0.242	0.952
Protein S [%]	0.119		-0.267; 0.506	0.540		0.035	-0.385; 0.454	0.869
Quick value [%]	-0.047		-0.186; 0.092	0.499		-0.023	-0.170; 0.125	0.758
INR^e^	0.010		-0.100; − 0.121	0.851		-0.016	-0.134; 0.101	0.786

^a^ After exclusion of individuals with anticoagulation therapy (n = 29); ^b^ dependent variables: blood coagulation markers, exposure variable: Fatty Liver Index, adjusted for age, alcohol consumption, sex, education, smoking and physical activity; ^c^ dependent variables: blood coagulation markers, exposure variable: Fatty Liver Index, additionally adjusted for stroke, hypertension, myocardial infarction, non HDL cholesterol ^d^ activated partial thromboplastin time; ^e^ international normalized ratio, INR times 100

## Discussion

The findings of the present study clearly show statistically significant associations between the FLI and a range of blood coagulation markers. All hemostatic factors and clotting tests (except for aPTT) were affected by increasing liver fat content.

In their study, Hörber et al. [22] followed a similar approach, and the coagulation markers tested in relation to fatty liver showed the same trends as those found in our analyses. They demonstrated that fatty liver is significantly positively associated with protein C, protein S, fibrinogen, and factor VIII and negatively associated with antithrombin III and aPTT. According to their interpretation, a fatty liver leads to an increased synthesis of proteins C and S in the liver, with protein S being a coproduct of protein C. As natural anticoagulants, they inhibit the coagulation cascade, which would counteract a prothrombotic state promoted by fatty liver [23, 24]. This hypothesis is supported by our data since both proteins were positively associated with an increasing FLI. As in our analyses, Hörber et al. also reported increased fibrinogen and factor VIII levels with increasing liver steatosis. Fibrinogen is a glycoprotein that is produced in the liver and contributes to the formation of a thrombus at the end of blood clotting [25]. Factor VIII is produced not only in the hepatocytes of the liver but also in the kidneys, lymphatic tissue, and endothelial cells [26]. Hörber et al. [22] also reported a negative association between antithrombin III, aPTT, and fatty liver, which was confirmed by our study results. Since antithrombin III is a natural anticoagulant produced in the liver and inhibits numerous clotting factors, reduced production with increasing FLI leads to increased interference with the coagulation cascade. This may eventually increase the risk of thrombosis and subsequently of other diseases [27]. aPTT is a coagulation test based on partial thromboplastin time and is used clinically to indicate abnormalities within the coagulation system [11]. An elevated aPTT value indicates an increased bleeding tendency, and a low value indicates a decreased bleeding tendency [28]. aPTT reflects the clotting time of the intrinsic pathway, and a shortened clotting time and thus faster thrombus formation makes sense from a physiological point of view [29]. This effect is evident based on the present results.

Since only a few studies have investigated the relationship between FLI and blood coagulation markers, our results are compared to studies using NAFLD as an endpoint. It is therefore important to understand and explain the term “NAFLD”. According to the European Association for the Study of the Liver (EASL) [4], NAFLD is characterized by excessive hepatic fat accumulation (associated with insulin resistance) and defined by the presence of steatosis in more than 5% of hepatocytes in association with metabolic risk factors, particularly obesity and type 2 diabetes, and in the absence of excessive alcohol consumption or other chronic liver diseases. There are two types of NAFLD: non-alcoholic fatty liver (NAFL) and the progressive form non-alcoholic steatohepatitis (NASH), which is characterized by chronic inflammation and death of liver cells that can progress to fibrosis and even the most severe form of metabolic cirrhosis. People usually develop one or the other form of NAFLD, although the transition is fluid. Kotronen et al. [30] measured various coagulation factors in a retrospective study comparing subjects with and without previously diagnosed non-alcoholic fatty liver disease (NAFLD). Liver fat was visualized in their work by H-magnetic resonance spectroscopy (H-MRS). An increase in the coagulation factors examined was found for subjects with NAFLD, but D-dimer values remained almost unchanged. However, since D-dimers are formed physiologically as cleavage products during the dissolution of fibrin [31], an increased blood concentration, as seen in our study, can be expected (in parallel with increasing fibrinogen). D-dimer concentrations are clinically relevant when excessive blood clotting and/or thrombus formation is suspected and provide important information as to whether clots were actually formed (and dissolved) in the body [32, 33]. The assumption that an elevated FLI may cause a procoagulant or prothrombotic state is also supported by the findings of Orgestra et al. [34]. In their review article, they compared published reports of changes in coagulation parameters associated with NAFLD. They confirmed that endothelial vascular dysfunction, platelet abnormalities and alterations in factors involved in the coagulation cascade and fibrinolysis may contribute to a prothrombotic state in patients with NAFLD. At the same time, they noted that currently available data on the role of platelets and changes in the coagulation system are sparse and often contradictory, highlighting the growing need for larger, prospective studies with well-defined patient groups and comprehensive tests to assess the hemostatic profile. In their paper, Virovic et al. [35] confirm the above statement that, despite research efforts, the link between NAFLD and coagulation disorders has not yet been established, in part because of the inadequacy of the tests used to assess these disorders. They argue that the close association of NAFLD and metabolic syndrome makes it difficult to distinguish the extent to which the two entities contribute independently to a procoagulant state. However, they concluded that there is growing evidence that NAFLD and NASH in particular may contribute significantly and independently to a procoagulant and prothrombotic state, independent of the presence of other factors. Finally, they also argue for the urgent need for larger population-based studies with careful selection of the methods and factors studied to clarify the complex changes in the coagulation process that occur in vivo in NAFLD, their interactions and clinical outcomes. In our study, we used FLI for screening NAFDL, an approach that is easily applicable for screening a large number of subjects. Thus, the relationship between this index and markers of the coagulation system is important information for its future application.

Hörber et al. [15] showed in another study that early prevention through lifestyle intervention had a massive influence on changes in the blood coagulation system. They studied 100 individuals with impaired glucose tolerance or impaired fasting plasma glucose (i.e., prediabetic subjects) who participated in a one-year lifestyle intervention. In addition to precise metabolic phenotyping and MRS-based determination of liver fat content, they also conducted a comprehensive analysis of blood coagulation parameters before and after the intervention. They were able to establish a causal link between a decrease in liver fat content and an improvement in hemostasis and thus indicate a future preventive and therapeutic target. Improvement of the prothrombotic status by lifestyle modifications that lead to decreased FLI seems promising and should be regarded as a disease-preventive measure. Since the information for the calculation of the FLI can be collected easily and quickly by taking simple measurements available in everyday clinical practice and the correlation of the FLI with the development of various disease patterns could already be demonstrated, the FLI represents a simple tool for diagnostics. In a population-based cohort study using the UK Biobank database, Zou et al. [36] found that the FLI was not the best measure for detecting liver steatosis; however, the FLI was significantly associated with prevalent and incident cardiovascular diseases. Additionally, Chang-Hoon Lee et al. [37] reported that a higher FLI score increases the risk of all-cause mortality and the risk of myocardial infarction and stroke. They observed that a simple (repeated) assessment of NAFLD status based on FLI could help clinicians identify groups at higher risk for all-cause mortality, myocardial infarction and stroke. The association between improving (i.e., decreasing) FLI and the decrease in cardiovascular disease risk also supports the clinical benefits of NAFLD prevention and treatment. Thus, the reported associations between FLI and coagulation markers in the present study may indicate a mechanistic pathway that could partly mediate such effects.

The observed lack of association between FLI and coagulation factors in patients with diabetes confirms that the specific metabolic situation dominates over the enrichment of fat in the liver [35, 38]. Patients with a diagnosis of diabetes show an increased thrombotic risk mainly mediated by the presence of insulin resistance, dysglycemia, and an increased inflammatory state, among other alterations [39]. This prothrombotic state in diabetic subjects is related to an overall increased cardiovascular disease risk [40]. According to our results, the relationships between FLI and hemostatic factors are weakened in prediabetic subjects (versus normoglycemic subjects); however, as discussed above, lifestyle intervention may restore metabolic and blood clotting derangements [41].

It is well described that a prothrombotic state could be the consequence of malignancies when tumor cells activate the coagulation system [42]. Besides, NAFLD is closely associated with hepatic and recently with extrahepatic cancers, such as bladder cancer [43]. Our study participants did not suffer from acute malignancies at the time of blood collection; however, as for other serious diseases, former cancer cases were not excluded from this cross-sectional analysis.

### Study strengths and limitations

The strengths of our study are a large sample size, the population-based design, the availability of standardized data on cardiovascular risk factors, and standardized anthropometric and laboratory measurements. Possible confounders were available and could be considered as adjustment variables in the regression models. However, some limitations must be considered. As the analysis is based on the follow-up of a population-based study, it is possible that the participants are not representative of the original sample. It cannot be completely excluded that selection bias may have influenced the present results. Furthermore, future studies should also identify other existing liver diseases such as hepatitis to avoid false positive FLI results. The results cannot be related to people of other ethnic origins or age groups, as only study participants born in the period from 1945 to 1964 were included. Furthermore, an exact measurement of liver fat content (e.g., by MR imaging) is more precise but much more cost-intensive than the calculated FLI.

## Conclusion

Our results demonstrate a relationship between the FLI and blood clotting markers, with a high FLI being associated with a pro-coagulative state. Patients at high risk of developing conditions such as thrombosis, CVD or stroke may benefit from this simple and fast diagnostic measure. However, further population-based studies are needed to clarify the complex changes in the coagulation process associated with the fatty liver index and their interactions with other metabolic derangements and subsequent clinical outcomes. The FLI may serve as a screening test to identify subjects that should undergo precise characterization of the liver and suitable intervention.

## Data Availability

The data are subject to national data protection laws, and restrictions were imposed by the Ethics Committee of the Bavarian Chamber of Physicians to ensure data privacy of the study participants. Therefore, data cannot be made freely available in a public repository. However, data can be requested through an individual project agreement with KORA via the online portal KORA (https://epi.helmholtz-muenchen.de/).

## Abbreviations

aPTT: Activated thromboplastin time
EALS: European Association for the Study of the Liver
FLI: Fatty Liver Index
H-MRS: H-magnetic resonance spectroscopy
INR: International thromboplastin time
KORA: Cooperative Health Research in the region of Augsburg
MONICA: Monitoring trends and determinants in cardiovascular disease
MRS: Magnetic resonance spectroscopy
NAFLD: Non-alcoholic fatty liver disease
NASH: Non-alcoholic steatohepatitis

## References

[1] Younossi ZM, Koenig AB, Abdelatif D, Fazel Y, Henry L, Wymer M. Global epidemiology of nonalcoholic fatty liver disease-Meta-analytic assessment of prevalence, incidence, and outcomes. Hepatology [Internet]. 2016 [cited 2023 Apr 16];64:73–84. Available from: https://pubmed.ncbi.nlm.nih.gov/26707365/10.1002/hep.28431 26707365

[2] Berardo C, Di Pasqua LG, Cagna M, Richelmi P, Vairetti M, Ferrigno A. Nonalcoholic fatty liver disease and non-alcoholic steatohepatitis: current issues and future perspectives in preclinical and clinical research. Int J Mol Sci [Internet]. 2020 [cited 2023 Apr 14];21:1–30. Available from: /pmc/articles/PMC7766139/. 10.3390/ijms21249646 PMC7766139 33348908

[3] Eslam M, Newsome PN, Sarin SK, Anstee QM, Targher G, Romero-Gomez M et al. A new definition for metabolic dysfunction-associated fatty liver disease: An international expert consensus statement. J Hepatol [Internet]. 2020 [cited 2023 Apr 14];73:202–9. Available from: https://pubmed.ncbi.nlm.nih.gov/32278004/10.1016/j.jhep.2020.03.039 32278004

[4] Marchesini G, Day CP, Dufour JF, Canbay A, Nobili V, Ratziu V et al. EASL-EASD-EASO Clinical Practice Guidelines for the management of non-alcoholic fatty liver disease. J Hepatol [Internet]. 2016 [cited 2023 Apr 14];64:1388–402. Available from: http://www.journal-of-hepatology.eu/article/S0168827815007345/fulltext 10.1016/j.jhep.2015.11.004 27062661

[5] Geier A, Tiniakos Di, Denk H, Trauner M. From the origin of NASH to the future of metabolic fatty liver disease. Gut [Internet]. 2021 [cited 2023 Apr 14];70:1570. Available from: /pmc/articles/PMC8292567/10.1136/gutjnl-2020-323202 PMC8292567 33632710

[6] Singh S, Allen AM, Wang Z, Prokop LJ, Murad MH, Loomba R. Fibrosis progression in nonalcoholic fatty liver vs nonalcoholic steatohepatitis: a systematic review and meta-analysis of paired-biopsy studies. Clin Gastroenterol Hepatol [Internet]. 2015 [cited 2023 Jun 14];13:643–654.e9. Available from: https://pubmed.ncbi.nlm.nih.gov/24768810/10.1016/j.cgh.2014.04.014 PMC4208976 24768810

[7] Idilman IS, Ozdeniz I, Karcaaltincaba M (2016). Hepatic steatosis: etiology, patterns, and quantification. Seminars in Ultrasound. CT and MRI.

[8] Bedogni G, Bellentani S, Miglioli L, Masutti F, Passalacqua M, Castiglione A et al. The fatty liver index: a simple and accurate predictor of hepatic steatosis in the general population. BMC Gastroenterol. 2006;6. 10.1186/1471-230X-6-33 PMC1636651 17081293

[9] Younossi ZM, Loomba R, Rinella ME, Bugianesi E, Marchesini G, Neuschwander-Tetri BA et al. Current and Future Therapeutic Regimens for Nonalcoholic Fatty Liver Disease and Nonalcoholic Steatohepatitis. Hepatology [Internet]. 2018 [cited 2023 Jun 5];68:361. Available from: /pmc/articles/PMC6508084/10.1002/hep.29724 PMC6508084 29222911

[10] Papatheodoridi M, Cholongitas E (2019). Diagnosis of non-alcoholic fatty liver Disease (NAFLD): current concepts. Curr Pharm Des.

[11] Kurtz A, Pape H-C, Silbernagl S, Bondke Persson A, Brenner B, Burckhardt G et al. Physiologie [Internet]. Stuttgart: Georg Thieme Verlag; 2018 [cited 2022 Dec 29]. Available from: https://eref.thieme.de/10.1055/b-006-149284

[12] Northup PG, Caldwell SH. Coagulation in liver disease: a guide for the clinician. Clin Gastroenterol Hepatol [Internet]. 2013 [cited 2023 Apr 18];11:1064–74. Available from: https://pubmed.ncbi.nlm.nih.gov/23506859/10.1016/j.cgh.2013.02.026 23506859

[13] Chaudhry R, Babiker HM, Physiology StatPearls. 2018 [cited 2022 Jul 15]. Available from: http://www.ncbi.nlm.nih.gov/pubmed/29489185

[14] Armandi A, Rosso C, Caviglia GP, Bugianesi E. Insulin Resistance across the Spectrum of Nonalcoholic Fatty Liver Disease. Metabolites [Internet]. 2021 [cited 2023 Apr 14];11. Available from: https://pubmed.ncbi.nlm.nih.gov/33800465/10.3390/metabo11030155 PMC8000048 33800465

[15] Hörber S, Lehmann R, Fritsche L, Machann J, Birkenfeld AL, Häring HU (2021). Lifestyle intervention improves prothrombotic Coagulation Profile in individuals at high risk for type 2 diabetes. J Clin Endocrinol Metab.

[16] Kirchberger I, Meisinger C. KORA-Studie: Ergebnisse aus 20 Jahren Gesundheitsforschung in Augsburg. Public Health Forum. 2012;20:19.e1-19.e3.

[17] Löwel H, Döring A, Schneider A, Heier M, Thorand B, Meisinger C. The MONICA Augsburg surveys - basis for prospective cohort studies. Gesundheitswesen. Gesundheitswesen; 2005. 10.1055/s-2005-858234 16032512

[18] World Health Organization. Obesity: preventing and managing the global epidemic : report of a WHO consultation. World Health Organization; 2000. 11234459

[19] Zahn K, Linseisen J, Heier M, Peters A, Thorand B, Nairz F et al. Body fat distribution and risk of incident ischemic stroke in men and women aged 50 to 74 years from the general population. The KORA augsburg cohort study. PLoS ONE. 2018;13. 10.1371/journal.pone.0191630 PMC5798769 29401461

[20] Meisinger C, Thorand B, Schneider A, Stieber J, Döring A, Löwel H (2002). Sex differences in risk factors for incident type 2 diabetes mellitus: the MONICA Augsburg Cohort study. Arch Intern Med.

[21] World Health Organisation. WHO Expert Committee on Biological Standardization. Guidelines forthromboplastins and plasma used to control oral anticoagulant therapy. 1999.

[22] Hörber S, Lehmann R, Stefan N, Machann J, Birkenfeld AL, Wagner R et al. Hemostatic alterations linked to body fat distribution, fatty liver, and insulin resistance. Mol Metab. 2021;53. 10.1016/j.molmet.2021.101262 PMC8165974 34082137

[23] Dahlbäck B, Villoutreix BO. Molecular recognition in the protein C anticoagulant pathway. J Thromb Haemostasis J Thromb Haemost; 2003. p. 1525–34. 10.1046/j.1538-7836.2003.00299.x 12871288

[24] Rezende SM, Simmonds RE, Lane DA. Coagulation, inflammation, and apoptosis: different roles for protein S and the protein S-C4b binding protein complex. Blood Blood; 2004. p. 1192–201. 10.1182/blood-2003-05-1551 12907438

[25] De Moerloose P, Casini A, Neerman-Arbez M (2013). Congenital fibrinogen disorders: an update. Semin Thromb Hemost.

[26] Mazurkiewicz-Pisarek A, Płucienniczak G, Ciach T, Plucienniczak A. The factor VIII protein and its function. Acta Biochim Pol Polskie Towarzystwo Biochemiczne; 2016. p. 11–6. 10.18388/abp.2015_1056 26824291

[27] Kim H, Kim H, Park JH, Kim YH, Oh SJ, Suh BJ (2017). Alcohol consumption, high-density lipoprotein cholesterol, Antithrombin III, and body Mass Index are Associated with Great Saphenous Vein Reflux in the thigh. Ann Vasc Surg.

[28] Tagariello G, Radossi P, Salviato R, Zardo M, De Valentin L, Basso M et al. Clinical relevance of isolated prolongation of the activated partial thromboplastin time in a cohort of adults undergoing surgical procedures. Blood Transfusion [Internet]. 2017 [cited 2023 Apr 16];15:557. Available from: /pmc/articles/PMC5649965/10.2450/2016.0047-16 PMC5649965 27483477

[29] Tripodi A, Chantarangkul V, Martinelli I, Bucciarelli P, Mannucci PM. A shortened activated partial thromboplastin time is associated with the risk of venous thromboembolism. Blood [Internet]. 2004 [cited 2023 Apr 16];104:3631–4. Available from: https://ashpublications.org/blood/article/104/12/3631/89062/A-shortened-activated-partial-thromboplastin-time 10.1182/blood-2004-03-1042 15297315

[30] Kotronen A, Joutsi-Korhonen L, Sevastianova K, Bergholm R, Hakkarainen A, Pietiläinen KH et al. Increased coagulation factor VIII, IX, XI and XII activities in non-alcoholic fatty liver disease. Liver Int [Internet]. 2011 [cited 2023 Mar 9];31:176–83. Available from: https://pubmed.ncbi.nlm.nih.gov/21134109/10.1111/j.1478-3231.2010.02375.x 21134109

[31] Folsom AR, Delaney JAC, Lutsey PL, Zakai NA, Jenny NS, Polak JF (2009). Associations of factor VIIIc, D-dimer, and plasmin-antiplasmin with incident cardiovascular disease and all-cause mortality. Am J Hematol.

[32] Akgul O, Uyarel H (2013). D-dimer: a novel predictive marker for cardiovascular disease. Int J Cardiol.

[33] di Castelnuovo A, de Curtis A, Costanzo S, Persichillo M, Olivieri M, Zito F (2013). Association of D-dimer levels with all-cause mortality in a healthy adult population: findings from the MOLI-SANI study. Haematologica.

[34] Ogresta D, Mrzljak A, Berkovic MC, Bilic-Curcic I, Stojsavljevic-Shapeski S, Virovic-Jukic L. Coagulation and Endothelial Dysfunction Associated with NAFLD: Current Status and Therapeutic Implications. J Clin Transl Hepatol [Internet]. 2022 [cited 2023 Apr 16];10:339–55. Available from: https://pubmed.ncbi.nlm.nih.gov/35528987/10.14218/JCTH.2021.00268 PMC9039716 35528987

[35] Virović-Jukić L, Stojsavljević-Shapeski S, Forgač J, Kukla M, Mikolašević I. Non-alcoholic fatty liver disease - a procoagulant condition? Croat Med J [Internet]. 2021 [cited 2023 Apr 16];62:25–33. Available from: https://pubmed.ncbi.nlm.nih.gov/33660958/10.3325/cmj.2021.62.25 PMC7976878 33660958

[36] Zou B, Yeo YH, Cheung R, Ingelsson E, Nguyen MH (2021). Fatty Liver Index and Development of Cardiovascular Disease: findings from the UK Biobank. Dig Dis Sci.

[37] Lee CH, Han K, Do, Kim DH, Kwak MS. The repeatedly elevated fatty liver index is Associated with increased mortality: a Population-Based Cohort Study. Front Endocrinol (Lausanne). 2021;12. 10.3389/fendo.2021.638615 PMC7996574 33776934

[38] Chalasani N, Younossi Z, Lavine JE, Diehl AM, Brunt EM, Cusi K et al. The diagnosis and management of non-alcoholic fatty liver disease: Practice guideline by the American Gastroenterological Association, American Association for the Study of Liver Diseases, and American College of Gastroenterology. Gastroenterology [Internet]. 2012 [cited 2023 Apr 16];142:1592–609. Available from: http://www.gastrojournal.org/article/S0016508512004945/fulltext 10.1053/j.gastro.2012.04.001 22656328

[39] Hess K. The vulnerable blood. Hamostaseologie [Internet]. 2015 [cited 2023 Apr 3];35:25–33. Available from: http://www.ncbi.nlm.nih.gov/pubmed/29589350 10.5482/HAMO-14-09-0039 29589350

[40] Huang D, Refaat M, Mohammedi K, Jayyousi A, Al Suwaidi J, Abi Khalil C. Macrovascular Complications in Patients with Diabetes and Prediabetes. Biomed Res Int [Internet]. 2017 [cited 2023 Apr 16];2017. Available from: /pmc/articles/PMC5697393/10.1155/2017/7839101 PMC5697393 29238721

[41] Alsharidah AS. Diabetes mellitus and diabetic nephropathy: a review of the literature on hemostatic changes in coagulation and thrombosis. Blood Res [Internet]. 2022 [cited 2023 Apr 3];57:101–5. Available from: https://pubmed.ncbi.nlm.nih.gov/35620906/10.5045/br.2022.2021204 PMC9242838 35620906

[42] Caine GJ, Stonelake PS, Lip GYH, Kehoe ST. The Hypercoagulable State of Malignancy: Pathogenesis and Current Debate. Neoplasia [Internet]. 2002 [cited 2023 Jun 14];4:465. Available from: /pmc/articles/PMC1550339/10.1038/sj.neo.7900263 PMC1550339 12407439

[43] Crocetto F, Pandolfo SD, Aveta A, Martino R, Trama F, Caputo VF et al. A Comparative Study of the Triglycerides/HDL Ratio and Pseudocholinesterase Levels in Patients with Bladder Cancer. Diagnostics [Internet]. 2022 [cited 2023 Jun 14];12:431. Available from: https://www.mdpi.com/2075-4418/12/2/431/htm. 10.3390/diagnostics12020431 PMC8871224 35204522

